# Ultrasound-treated water-dispersible myofibrillar protein microgel particles: An effective strategy for stable Pickering emulsion gels with enhanced interfacial properties, dysphagia-oriented texture modification and improved encapsulation capacity^☆^

**DOI:** 10.1016/j.ultsonch.2026.107972

**Published:** 2026-07-22

**Authors:** Linmeng Wang, Ke Li, Yu Wang, Wuchao Ma, Jianan Yan, Ge Han, Haitao Wu, Yanhong Bai

**Affiliations:** a Key Laboratory of Cold Chain Food Processing and Safety Control, Ministry of Education, College of Food and Bioengineering, Zhengzhou University of Light Industry, Ke Xue Road No. 136, Zhengzhou 450001, PR China; b SKL of Marine Food Processing & Safety Control, National Engineering Research Center of Seafood, Collaborative Innovation Center of Seafood Deep Processing, Key Laboratory of Aquatic Product Processing and Quality Control, School of Food Science and Technology, Dalian Polytechnic University, Dalian 116034, PR China; c College of Food Science and Engineering, Henan University of Technology, Zhengzhou 450001, PR China

**Keywords:** PSE, Ultrasound, Microgel particle, Emulsion gel, Curcumin, Encapsulation

## Abstract

To improve the poor processing adaptability and weak interfacial activity of pale, soft and exudative (PSE)-like chicken breast meat myofibrillar protein (MP) under low-salt conditions, this study proposed a colloidal restructuring strategy. PSE-like MP was diluted with water and subjected to heating to form aggregates. These aggregates were converted into water-dispersible MP microgel particles (MMPs) by ultrasound treatment and used to prepare Pickering emulsion gels for developing meat protein-based dysphagia foods. The influence of ultrasound time (20 kHz, 450 W, 0–25 min) on the structural and interfacial properties of water-dispersible MMPs was investigated, and the stability of Pickering emulsions was examined, along with the gel properties, dysphagia‑oriented texture, and encapsulation capacity of the emulsion gels. Results showed that moderate ultrasound reduced particle size, altered secondary structure, and enhanced oil–water interfacial adsorption by increasing surface hydrophobicity and improving wettability. Ultrasound treatment for 20 min showed the best performance, increasing the MMP contact angle to 75.70° and interfacial adsorbed protein content to 49.18%, producing smaller droplets and greater stability. A compact gel network was formed, which improved gel performance and curcumin encapsulation efficiency. IDDSI tests showed that ultrasound improved shape stability and crushability, shifting the emulsion gels classification from Level 4 to Level 5. These findings indicate that ultrasound modified PSE-like MP into interfacially active water-dispersible microgel particles, imparting stable emulsion gels and forming meat protein-based swallowing-friendly systems with nutritional encapsulation of active substances. This approach offers guidance for processing and utilizing PSE‑like muscle protein in high‑protein dysphagia food development.

## Graphic abstract

**Figure:**
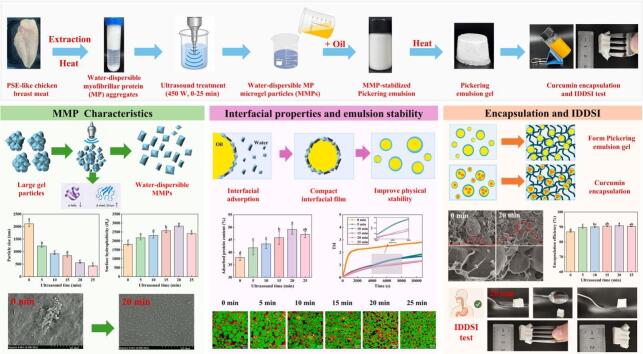

## 1 Introduction

The global demographic transition toward an older population is accelerating, with the number of individuals aged 65 and above projected to approach one billion by 2030 [1]. This shift has led to a concurrent increase in both dysphagia prevalence and associated nutritional shortcomings. Texturally adapted foods are routinely prescribed to manage bolus flow and minimize aspiration risk in those with swallowing disorders [2]. However, most dysphagia diets still rely on hydrocolloid thickeners for viscosity enhancement, which may compromise protein supplementation [3]. Elderly individuals need at least 1.2 g/kg/day of protein to preserve skeletal muscle mass and sustain immune competence [4]. Meat serves as a vital source of high-quality protein, yet the tough fibrous structure of intact meat limits its direct use in foods for older adults and individuals with dysphagia. Although pale, soft, and exudative (PSE)-like chicken meat is generally regarded as a quality defect, it still retains nutrients such as protein, zinc, B vitamins, and essential amino acids, and also shows good digestibility [5]. The applications of PSE-like chicken meat have been largely confined to low-value-added processing, without fully exploiting its structural tunability as a protein matrix [6]. Due to its relatively low pH and partial protein denaturation, PSE-like chicken meat exhibits poor processing functionality and tends to form loose and soft gel structures [7]. These characteristics align well with the dual requirements of dysphagia-oriented foods for soft texture and high-protein nutrition.

As the major salt‑soluble protein in muscle, myofibrillar protein (MP) accounts for more than half of the total protein, mainly determines gelation, emulsification, and water retention in meat products, but its functionality strongly depends on ionic strength [8]. Although NaCl improves MP solubilization and processing performance, excessive salt intake is associated with hypertension and cardiovascular risks in older adults. Under low-salt conditions, MP shows poor solubility and strong intermolecular aggregation, leading to weak interfacial activity and limited performance in emulsion and gel systems [9]. Earlier studies have largely addressed the suppression of MP aggregation under low-salt or salt-free conditions to enhance solubility, or promoting gelation [10], whereas relatively limited attention has been paid to exploiting its intrinsic aggregation tendency and thermosensitive behavior of MP as structural advantages. Therefore, whether this characteristic can be exploited to restructure low-salt MP into interfacially active colloidal particles and construct novel stable systems warrants further investigation.

Pickering emulsion gels have attracted increasing attention for dysphagia-oriented foods. They rely on the interfacial adsorption of solid particles to form a protective layer. Meanwhile, the synergistic interplay among the interfacial particle layer, oil droplets, and the continuous gel network further enhances emulsion and gel stability [11]. Among food-grade solid particles, protein microgel particles are particularly attractive because of their natural origin, nutritional value, deformability, and tunable structure [12]. Plant and whey protein microgel particles have been extensively studied and applied in emulsion stabilization, fat replacement, and bioactive compound delivery [13], [14]. In contrast, meat protein microgel particles exhibit unique advantages in enhancing the cohesiveness, lubricity, and textural adaptability of Pickering emulsion gels [15], showing greater potential for dysphagia‑oriented food development. However, existing research has largely centered on preparation methods and fundamental functional evaluation of muscle protein microgel particles, while exploration of their application expansion remains relatively limited. In particular, the preparation of muscle protein microgel particles in water‑dispersible systems and their stabilizing effects on Pickering emulsion gels remain unclear.

Ultrasound serves as a green processing technique for modulating protein functionality through cavitation, microjets, turbulence, and shear forces [16]. Previous studies have shown that ultrasound can disrupt MP filamentous assembly and promote its stable dispersion in water [9]. Therefore, ultrasound may be suitable for converting heat-induced PSE-like MP aggregates into water-dispersible microgel particles. However, it remains unclear how ultrasound-induced structural changes in water-dispersible PSE-like MMPs govern their interfacial activity, Pickering emulsion stability, and emulsion gel functionality.

Curcumin, a naturally occurring lipophilic polyphenol, exerts anti-inflammatory, antioxidant, and gut microbiota-regulating effects, thereby holding considerable potential for ameliorating chronic health conditions in elderly populations [17]. Nevertheless, poor stability limits its oral bioavailability. The structured oil phase and stabilized interfaces of Pickering emulsion gels can provide potential microenvironments for encapsulating hydrophobic nutrients and bioactive compounds [18], which can enhance the loading stability of curcumin in food systems. However, it remains unclear whether water‑dispersible PSE-like MMP-stabilized emulsion gels are suitable as nutritional carriers for dysphagia populations.

In this study, PSE-like MP was diluted with water and subjected to heat-induced aggregation to form aggregates, which were then converted into water-dispersible MP microgel particles (MMPs) by ultrasound treatment. The effects of ultrasound time (20 kHz, 450 W, 0–25 min) on MMP structural and interfacial properties were investigated, and their roles in regulating Pickering emulsion stability, emulsion gel performance, dysphagia-oriented texture, and curcumin encapsulation were further evaluated. This study aimed to establish the relationship between ultrasound-induced colloidal restructuring, MMP interfacial functionality, and Pickering emulsion gel formation. The findings offer a structural design pathway for creating healthy, protein-rich foods suitable for dysphagia.

## 2 Materials and methods

### 2.1 Materials

Chicken breast meat exhibiting PSE-like characteristics (L* > 53 and pH_24 h_ < 5.7) served as the starting material and was pretreated following established procedures [5]. The selected samples were sealed in polyethylene bags to prevent moisture loss and contamination during storage and kept at − 18 °C for up to two weeks before use.

Edible soybean oil was purchased from Yihai Kerry Food Co., Ltd. (Shanghai, China). Analytical-grade reagents were utilized throughout this study.

### 2.2 Extraction of MP and preparation of MMP

Extraction of MP from PSE-like chicken breast meat followed the protocol of Li, et al. [19]. MP concentration was quantified via the Biuret method [20]. The MP (72–78 mg/mL per extraction) was diluted to 20 mg/mL with deionized water, then heat-treated, stored overnight, and homogenized as previously described [21]. Subsequently, 70 mL of the homogenized dispersion was transferred to a 150 mL beaker and subjected to ultrasonication using a Vibra Cell™ ultrasonic processor (VC750, Sonics & Materials, Inc., USA). The probe (13-mm diameter) was immersed to a depth of approximately 2.5 cm below the liquid surface. Ultrasonication was carried out at 20 kHz with an output power of 450 W for 0, 5, 10, 15, 20, and 25 min in pulse mode (2 s on/2 s off). The sample temperature was controlled below 20 °C, yielding an ultrasound intensity of 34.10 ± 0.60 W/cm^2^ [22]. The MMP dispersion obtained after ultrasound treatment was kept refrigerated at 4 °C for up to 24 h before use. The 0 min treatment group served as the control, representing the non‑ultrasonicated MP large gel particles.

### 2.3 Characterization of MMP

#### 2.3.1 Particle precipitation rate

Particle precipitation was assessed following the method of Wang, et al. [23]. Approximately 15 mL of each MP large gel particle/MMP dispersion was placed into a transparent glass container (inner diameter 2.8 cm, 20 mL) and maintained for 5 days under undisturbed conditions (4 °C). Supernatant layer height (H_1_) and total suspension height (H_0_) were measured every 24 h using a vernier caliper with an accuracy of 0.02 mm. The sedimentation index (%) was calculated as follows:

$$Particle\;precipitation\;rate\;\left(\%\right)\;=\frac{H_{1}}{H_{0}}\times 100$$

#### 2.3.2 Particle size and zeta potential

The MP large gel particle/MMP dispersions were diluted, and the particle size and zeta potential were determined following the method described previously [24].

#### 2.3.3 Microstructure

The surface morphology of the dried samples was examined by field-emission scanning electron microscopy (SEM; Regulus 8100, Hitachi Ltd., Tokyo, Japan). The dried powder samples were fixed onto specimen stubs using a double-sided conductive adhesive. After sputter-coating with gold, the dried powders were observed under 5000 × and 5 kV conditions [25].

#### 2.3.4 Secondary structure

The lyophilized sample was ground in an agate mortar, subsequently mixed with KBr to ensure homogeneity, and then compressed into a uniform thin sheet. The sheet was then subjected to Fourier transform infrared spectroscopy (FTIR) analysis using a spectrometer (Vertex 70, Bruker, Germany) over a wavelength range of 4000 to 400 cm^−1^. The obtained FTIR spectra were fitted to determine the relative contents of protein secondary structures [5].

#### 2.3.5 Surface hydrophobicity

The surface hydrophobicity of samples was analyzed following Sun, et al. [21] with minor adjustments. Prior to measurement, the MP large gel particle/MMP dispersion was diluted to 0.2 mg/mL with deionized water. Then, 10 μL of ANS solution (8 mM) was added to 2 mL of each diluted dispersion, and the mixture was incubated in the dark (10 min) to allow equilibrium binding between the probe and the exposed hydrophobic patches. The fluorescence intensities of ANS–dispersion conjugates were measured using a fluorescence spectrophotometer (F-7000, Hitachi, Tokyo, Japan) at excitation/emission wavelengths of 380/400–600 nm. The fluorescence intensity at the peak was used as an empirical indicator for evaluating relative changes in the surface hydrophobicity of the proteins.

#### 2.3.6 Three-phase contact angle

The sample powders obtained after freeze-drying for 48 h were compressed into thin sheets (1.5 mm in thickness and 12 mm in diameter). Each sheet was placed on the sample stage of a Tracker S interfacial rheometer (Teclis Scientific, France). A syringe deposited ultrapure water (5 μL) onto the sheet sample surface. After stabilization, the droplet profile was captured using a high-speed camera, and the contact angle was determined by circle-fitting [26].

### 2.4 Characterization of MMP-stabilized Pickering emulsion

Pickering emulsions were formed by mixing the MP large gel particle/MMP dispersions (prepared with different ultrasound times) with soybean oil (50%, v/v) and homogenizing at 10,000 rpm for 2 min.

#### 2.4.1 Emulsion particle size and zeta potential

The particle size and distribution of the emulsions were determined using a laser diffraction particle size analyzer (LS13320/ULM2, Beckman Coulter, USA) as described by Zhang, et al. [27]. The volume‑weighted mean diameter (d_4_,_3_) was adopted for droplet size reporting. Zeta potential measurements were performed as described in Section 2.3.2 at 25 °C after a 120 s equilibration period.

#### 2.4.2 Interfacial adsorbed protein content (AP)

Interfacial AP was quantified following the method of Lu, et al. [8]. Emulsion samples (2 mL) were centrifuged (10,000 × g, 10 min, 20 °C), and the protein concentration of the recovered aqueous phase (C_1_) was measured. The protein concentration of the original emulsion was defined as C_0_. AP was calculated as:

$$AP\;\left(\%\right)=\frac{{C_{0}-C}_{1}}{C_{0}}\times 100$$

#### 2.4.3 Interfacial tension

Soybean oil was mixed with 3% chromatographic adsorbent and stirred for 3 h until homogeneous. After centrifugation (10,000 × g, 30 min), the supernatant was harvested from the mixture. This entire step was repeated once. The resulting supernatant was subsequently mixed with 6% chromatographic adsorbent and stirred overnight. After a second centrifugation on the next day, the supernatant was recovered as purified soybean oil. The purified soybean oil was stored at 4 °C until further use [28].

For interfacial tension measurements, the MP large gel particle/MMP dispersion (1 mg/mL) was loaded into a syringe, and air bubbles were expelled. The syringe was then mounted on the sample holder of the interfacial rheometer, with its needle tip immersed in purified soybean oil and its position was adjusted with the aid of the image displayed on the computer screen. Droplets were formed at the tip by adjusting the downward displacement of the needle, and the dispersion was slowly injected into the oil phase at a rate of 25 μL per drop. Interfacial tension was recorded at 25 °C for 10,800 s. At least three consecutive measurements were performed for each sample [29].

#### 2.4.4 Turbiscan Stability Index (TSI)

The TSI of the emulsions was assessed with a dispersion stability analyzer (Turbiscan Lab, Formulaction, France). A 20 mL portion of each emulsion was moved into a sample bottle (bubble formation was avoided) and then subjected to 433 scans from the bottom to the top, with the total measurement duration being 3 h 25 s at 25 °C [30].

#### 2.4.5 Dynamic rheological characteristics

Rheological characterization was performed using a DHR-1 rheometer (TA Instruments Inc., USA) following the method of Wang, Zhang et al. [5]. Before each measurement, the samples were equilibrated for 2 min at room temperature. Then the Pickering emulsion was loaded onto a parallel plate geometry (40 mm diameter, 1000 μm gap) and subjected to a temperature ramp from 20 to 90 °C at 2 °C/min. The exposed edge of the sample was covered with silicone oil to prevent water evaporation. Within the linear viscoelastic region (LVR), the storage (G′) and loss (G″) moduli were measured at a frequency (0.1 Hz) and a consistent strain (0.5%).

#### 2.4.6 Confocal Laser Scanning Microscopy (CLSM)

The distribution of proteins and oil droplets in the emulsions was visualized using confocal laser scanning microscopy (FV3000, Olympus, Japan) equipped with a 40 × objective lens. Prior to measurement, the emulsion samples were stained with Nile red and Nile blue (both at 0.1%, w/v) to selectively label the oil phase (excitation 488 nm) and protein phase (excitation 633 nm), respectively. The stained specimens were mounted on glass slides, sealed with cover slips, and examined under the microscope.

#### 2.4.7 Storage stability and freeze-thaw stability

Fresh emulsions were dispensed into sealed glass bottles (20 mL capacity) with minimal headspace to prevent water evaporation and oxidation during storage. One set was allocated for storage stability evaluation and kept at 4 °C for 0, 1, 3, 7, and 11 days. Another set was designated for freeze-thaw treatment according to a previously described method [24]. After each designated storage period or freeze-thaw cycle, samples were photographed using a smartphone camera (iPhone 14, Apple, Cupertino, CA, USA). To ensure consistent imaging conditions, black cardboard was placed beneath and behind the samples as a uniform background, and all photographs were taken under the same lighting conditions and at a fixed distance from the samples.

### 2.5 Characterization of Pickering emulsion gel

The preparation of MMPs-stabilized emulsion gels from different ultrasound treatments was the same as described in Section 2.2. The resulting gels were stored at 4 °C for subsequent analyses.

#### 2.5.1 Gel strength and emulsion stability

The gel strength of the emulsion gels was measured using a texture analyzer (TA-XT Plus, Stable Micro Systems, UK) equipped with a P/0.5 cylindrical probe. The measurement parameters were set according to the method described by Feng, et al. [31]. Cylindrical glass containers (diameter 25 mm, height 40 mm) were used, and the gels were filled to two-thirds of its height. The pre-test and post-test speeds were set to 2 mm/s, and the test speed was set to 1 mm/s, with six replicate measurements per sample.

The emulsion stability of the emulsion gels was characterized by water-holding capacity (WHC), which was determined according to the method described by Hu et al. [30]. Approximately 2–3 g of emulsion gel (recorded as M_1_) was placed in a centrifuge tube and centrifuged at 6000 × g for 10 min. After centrifugation, the gel was removed, its surface was blotted dry, and then reweighed (recorded as M_2_). WHC was calculated as:

$$WHC\;\left(\%\right)=\frac{M_{2}}{M_{1}}\times 100$$

#### 2.5.2 Low-field nuclear magnetic resonance (LF-NMR) and magnetic resonance imaging (MRI)

LF-NMR and MRI measurements were conducted using an NMR analyzer (Niumag Electric Co., Shanghai, China). Approximately 2 g of the emulsion gel was placed into a 1.5 mL centrifuge tube. Prior to measurement, the cap of the centrifuge tube was trimmed to allow it to fit inside the NMR tube. The relaxation time (T_2_) analysis was performed using the Carr-Purcell-Meiboom-Gill (CPMG) sequence at an ambient temperature of approximately 32°C and a frequency of 22 MHz. The measurement parameters were: a scan range of 0–10,000 ms, 32 scans, a scan interval of 9,000 ms, and 15,000 echoes. Proton density images of the emulsion gels were acquired using multiple-spin-echo (MSE) pulse sequences. The acquired images were processed using the built-in Osiris software for pseudo-color rendering to visualize water distribution [5].

#### 2.5.3 Cryo-scanning electron microscopy (cryo-SEM) of Pickering emulsion gel

The microstructure of the Pickering emulsion gels was visualized using cryo-scanning electron microscopy (Regulus 8100, Hitachi Ltd., Tokyo, Japan), according to the method described by Tao, et al. [32]. Morphological images were acquired at an accelerating voltage of 5 kV and magnifications of 2000 × and 10,000 × .

#### 2.5.4 Rheological characteristics

##### 2.5.4.1 Steady shear test

Steady shear measurements were performed according to Rahman, et al. [33]. The apparent viscosity (η) of the Pickering emulsion gels was recorded across a shear rate range of 1–1000 s^−1^ at 25 °C.

##### 2.5.4.2 Frequency sweep

To evaluate the viscoelastic behavior of the emulsion gels, G′, G″, and loss tangent (tan δ = G″/G′) were obtained over a frequency range of 0.1–10 Hz within the LVR (0.5% strain) [34].

##### 2.5.4.3 Large amplitude oscillation shear (LAOS) test

LAOS behavior was characterized by sweeping the shear strain from 0.1% to 100% at 1 Hz and 25 °C, with G′ and G″ recorded throughout [34].

#### 2.5.5 Texture profile analysis (TPA) and international dysphagia diets standardization initiative (IDDSI) test

The TPA of the emulsion gels were performed according to the previously described method of Bourne [35] with minor modifications, using a texture analyser equipped with a P/36R cylindrical probe. The pre-test and post-test speeds for gel (diameter 20 mm, height 20 mm) texture property testing were 5 mm/s and the test speed was 1 mm/s. The compression distance was 40%, with a trigger force of 5 g, and tests were repeated six times.

Suitability for dysphagia was evaluated according to the IDDSI framework, which classifies texture-modified foods and drinks into eight levels (0–7). Three tests were conducted as follows. For the spoon tilt test, a gel sample was placed on a spoon, which was then slowly tilted to observe whether the sample retained its shape or slid off. For the fork drip test, a gel sample was scooped with a fork, and its retention between the prongs and dripping behavior were observed. For the fork pressure test, the gel sample was cut into cubes of 15 mm × 15 mm × 15 mm. A fork was then placed on the sample, and gentle thumb pressure was applied to assess its deformation and degree of fracture [36].

### 2.6 Characterization of curcumin-loaded emulsion gel

Curcumin (1%, w/w) was incorporated into soybean oil and subjected to ultrasonication (20 kHz, 300 W, 10 min) to ensure complete dissolution [37]. The curcumin-loaded soybean oil was then used to replace the original soybean oil and processed into emulsion gel preparation as detailed in Section 2.5. During this process, the temperature maintenance at 80 °C was adjusted to 20 min. The freshly prepared emulsion gels were cooled and held at 4 °C (12 h) prior to further characterization.

#### 2.6.1 Encapsulation efficiency (EE)

The curcumin-loaded emulsion gels were diluted to an appropriate concentration with anhydrous ethanol. Centrifugation at 10,000 × g for 10 min isolated the supernatant. The supernatant was then diluted with ethanol, and measured for absorbance at 424 nm using a UV spectrophotometer (TU-1810, General Instrument, China) [14]. The EE of curcumin was computed as:

EE (%) =.C2C1×100

where C_1_ represents the total curcumin content initially added, and C_2_ represents the curcumin content retained in the emulsion gel samples.

### 2.7 Data analysis

Experiments were conducted a minimum of three times, with the results reported as the mean ± standard deviation, unless specified otherwise. One-way analysis of variance (ANOVA) followed by Duncan’s multiple-range test (*p* < 0.05) was performed using SPSS 22 (IBM software, NY, USA).

## 3 Results and discussion

### 3.1 MMP particle precipitation rate

**Fig. 1:**
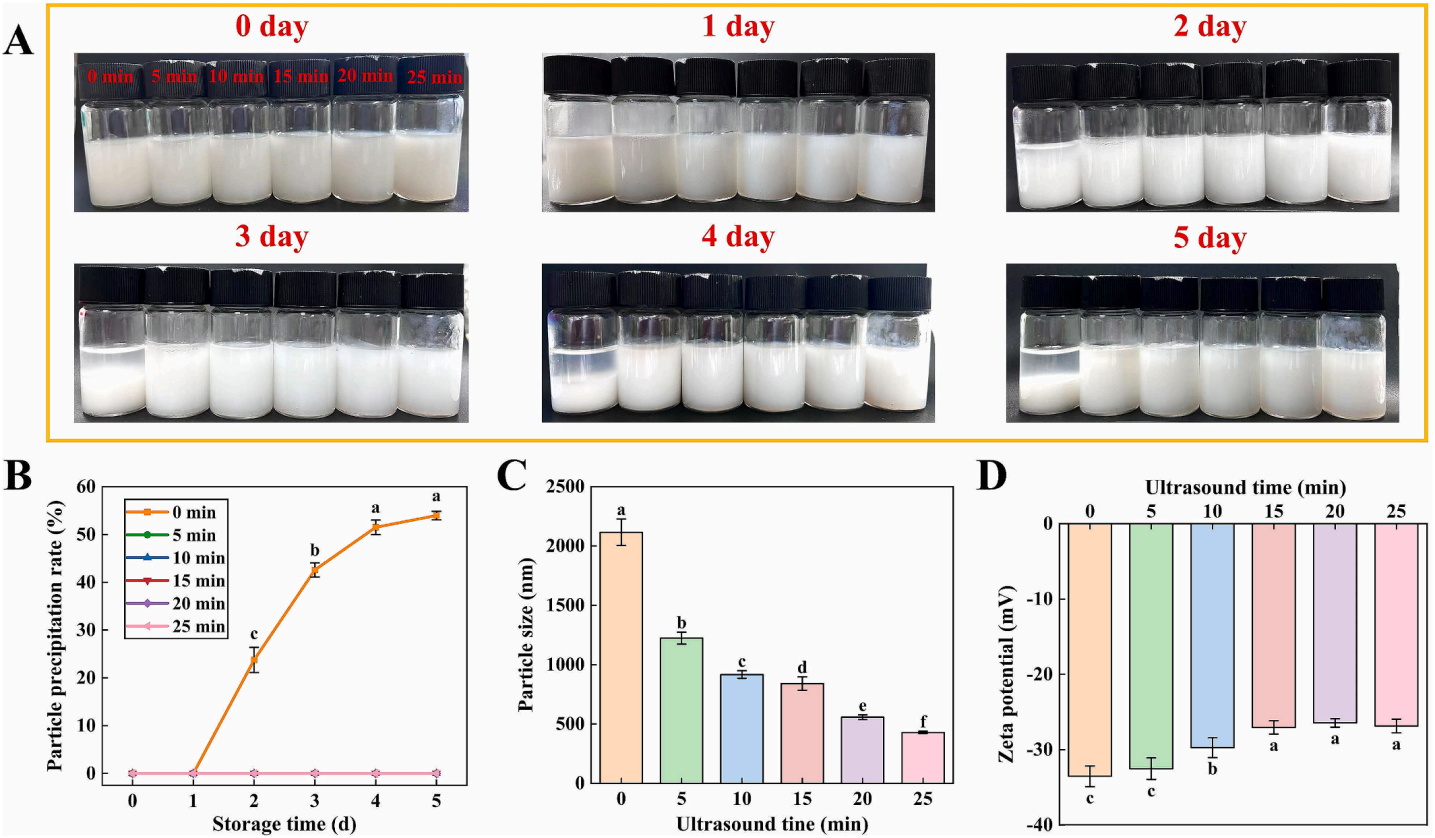
Photographs (A), particle precipitation rate (B), particle size (C) and zeta potential (D) of water-dispersible PSE-like myofibrillar protein microgel particles (MMPs) prepared with different ultrasound times (0, 5, 10, 15, 20, and 25 min). Different letters (a–f) above error bars indicate significant difference (*p* < 0.05) among different samples.

The precipitation rate of protein gel particles can be used to evaluate their stability and homogeneity during storage. The untreated large gel particles exhibited significant precipitation during storage. The precipitation rate increased significantly from day 2 to day 5 and reached 53.97 ± 0.89% on day 5 (Fig. 1B). After ultrasound treatment, negligible precipitation was observed in the gel particles throughout the 5-day storage period, regardless of the ultrasonication time. The macroscopic appearance further confirmed these results (Fig. 1A). In the untreated group, slight phase separation appeared at the top of the particle dispersion on day 2, and the separation became more pronounced with prolonged storage, accompanied by a clearer supernatant. In contrast, the ultrasonicated particle dispersions maintained good uniformity during storage, with no obvious phase separation or sediment deposition. The differences in precipitation behavior may mainly be attributed to particle size and solubility [23]. Larger particles are generally more prone to gravitational sedimentation, resulting in poorer dispersion stability. Ultrasound treatment effectively suppressed particle precipitation, probably by disrupting loosely bound aggregates, reducing the effective particle size, and improving dispersion uniformity. The underlying mechanisms require further verification.

### 3.2 MMP particle size and zeta potential

As shown in Fig. 1C, the particle size of MMPs decreased significantly from 2115.0 ± 111.9 nm to 428.9 ± 9.4 nm with increasing ultrasound time up to 25 min (*p* < 0.05), indicating that ultrasound effectively disrupted the large aggregated structures in MP gel particles. This reduction was mainly attributed to the transient high pressure, localized turbulence, and intense shear forces generated by ultrasound cavitation, which disrupted the non-covalent interactions maintaining protein aggregates and further promoted the disassembly and refinement of gel particles [38]. As the ultrasound time increased, the absolute zeta potential of MMPs decreased from − 33.53 ± 1.38 mV in the untreated group to − 26.45 ± 0.56 mV after 20 min of treatment, and then remained nearly unchanged at 25 min (Fig. 1D). This result indicates that ultrasound treatment reduced the apparent surface charge density of MMPs. However, this change did not compromise the dispersion stability. Therefore, the improved stability of ultrasonicated MMPs was mainly attributed to the reduction in particle size and the consequent weakening of gravitational sedimentation, rather than to electrostatic repulsion alone [9], [23]. The reduced zeta potential may be associated with ultrasound-induced disintegration of large aggregates and redistribution of charged groups on the particle surface [39].

### 3.3 MMP microstructure

**Fig. 2:**
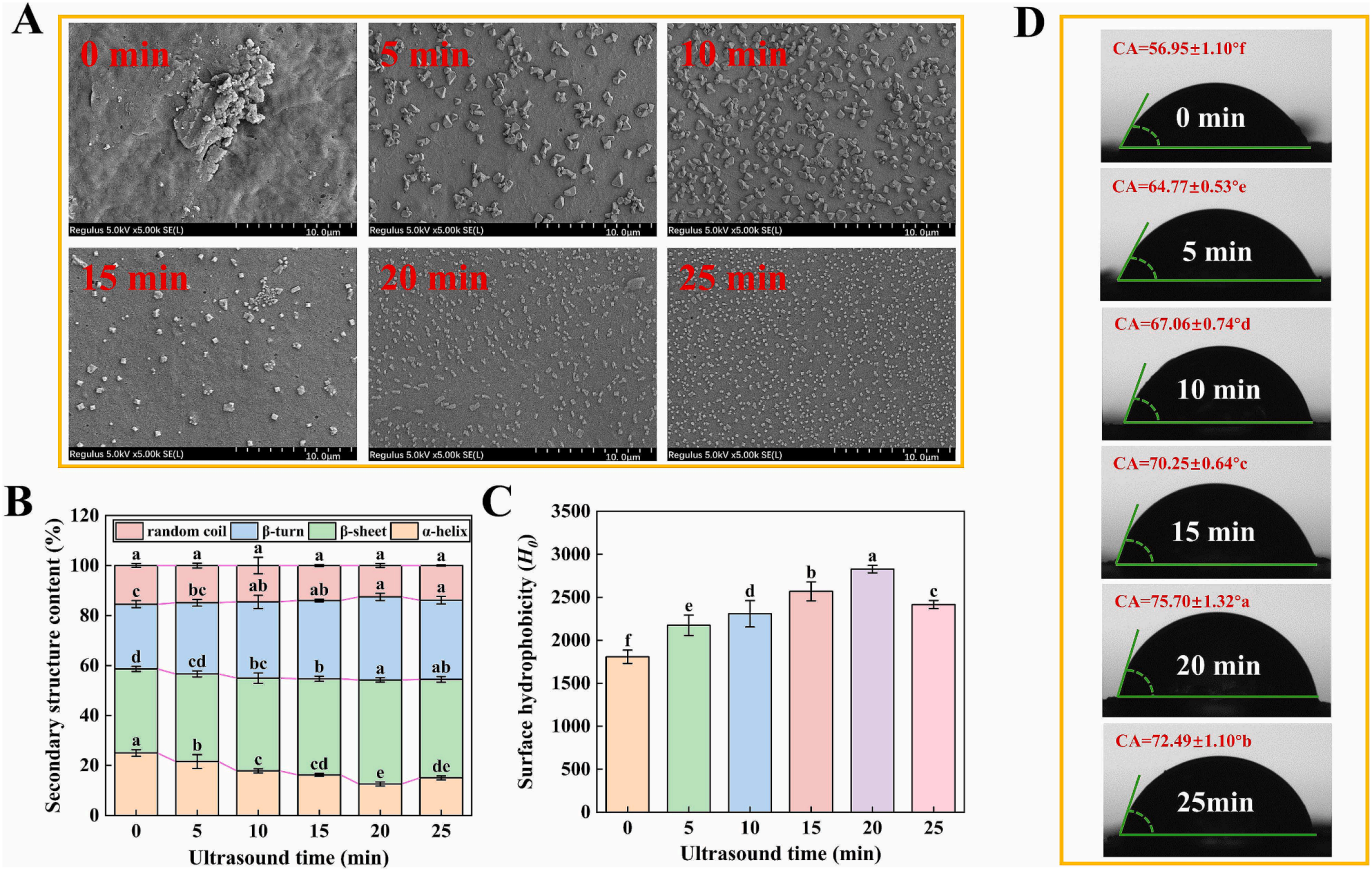
Microstructure (A), secondary structure (B), surface hydrophobicity (C) and three-phase contact angle (D) of water-dispersible PSE-like myofibrillar protein microgel particles (MMPs) prepared with different ultrasound times (0, 5, 10, 15, 20, and 25 min). Different letters (a–f) above error bars indicate significant difference (*p* < 0.05) among different samples.

SEM images further documented the regulatory influence of ultrasound treatment on MMP morphology and microstructure. The untreated large gel particles mainly exhibited large, stacked aggregates with irregular morphology and obvious agglomeration (Fig. 2A). After ultrasound treatment, the size of MMPs decreased markedly, and their morphology gradually changed from large agglomerated structures to smaller irregular polyhedral or fragmented gel particles. This confirms that ultrasound-induced cavitation, microjets, and shear forces effectively disrupted weakly connected regions within MMPs, further promoting the disassembly and refinement of large particles [38]. Upon prolonging the ultrasound treatment to 20–25 min, the MMPs became further refined and more uniformly distributed, while still retaining a rough and irregular surface morphology. In agreement with the particle size results, this further confirms that ultrasound promoted the disassembly, refinement, and surface remodeling of MMPs.

### 3.4 MMP secondary structure and surface hydrophobicity

As shown in Fig. 2B, ultrasound treatment markedly altered the secondary structure composition of MMPs. Relative to the untreated group, the α-helix content decreased from 25.00 ± 1.34% to 12.57 ± 0.81% after 20 min of ultrasound treatment, whereas the β-sheet and β-turn contents rose from 33.65 ± 0.98% and 25.90 ± 1.41% to 41.66 ± 0.83% and 33.22 ± 1.43%, respectively. After 25 min of ultrasound treatment, the α-helix content showed a slight rebound, while β-sheet and β-turn proportions marginally declined, however, these variations were not statistically distinguishable from those of the 20 min group. The stability of the α-helix conformation is mainly attributed to intramolecular hydrogen bonding. Therefore, its reduction suggests that moderate ultrasound-induced cavitation and mechanical shear disrupted part of the ordered conformation, leading to protein chain unfolding and rearrangement. Meanwhile, the increased β-sheet and β-turn contents reflected the conformational rearrangement of unfolded protein chains through intermolecular hydrogen bonding or hydrophobic interactions. These secondary structural changes may be associated with the exposure and redistribution of hydrophobic groups, thereby furnishing a structural basis for further conformational adjustment and particle adsorption at the oil–water interface [19]. However, when the ultrasound time was further extended, excessive cavitation may disturb the favorable conformational state formed by moderate unfolding, leading to partial rearrangement, refolding, or nonspecific aggregation of protein segments, thereby weakening their interfacial adaptability [40].

The surface hydrophobicity results further supported the above inference (Fig. 2C). The surface hydrophobicity of MMPs reached its maximum at 20 min of ultrasonication, followed by a decrease at 25 min. This implies that 20 min of ultrasound treatment facilitated protein unfolding, enhanced the surface exposure of hydrophobic residues originally embedded within the protein matrix [41]. The exposure of hydrophobic groups helped adjust the hydrophilic–hydrophobic balance of the particles, thereby providing favorable surface conditions for improved wettability and oil–water interfacial adsorption. Meanwhile, the changes in surface hydrophobicity were consistent with the particle size and zeta potential results. Ultrasound treatment reduced MMP particle size and increased the specific surface area, exposing more hydrophobic regions and surface groups to the aqueous environment. Concurrently, protein chain rearrangement and the exposure of hydrophobic domains may have led to the redistribution or partial shielding of charged groups, thereby reducing the apparent surface charge density. In contrast, excessive ultrasound caused the re‑burial or reduced exposure of hydrophobic regions on the particle surface [42].

### 3.5 MMP three-phase contact angle

The particle wettability is crucial for Pickering emulsion stabilization. This property is commonly characterized by the three-phase contact angle (θw/o), which determines emulsion type and particle retention at the interface. The θw/o values of all samples were below 90° (Fig. 2D), indicating their hydrophilic characteristics [21]. In general, a θw/o value closer to 90° favors particle adsorption and arrangement at the oil–water interface, thereby improving Pickering emulsion stability [29]. The θw/o values of the ultrasonicated MMPs were clearly higher than that of the untreated group (56.95 ± 1.10°), reaching a maximum value of 75.70 ± 1.32° after 20 min of ultrasound treatment. This indicates that moderate ultrasound treatment promoted hydrophobic group exposure and tuned the hydrophilic–hydrophobic balance of the particles toward a regime more conducive to interfacial adsorption. Under ultrasound treatment for 25 min, the slight decrease in θw/o values agreed with the surface hydrophobicity data, indicating that excessive ultrasound weakened the favorable wettability of MMPs.

### 3.6 Pickering emulsion particle size and zeta potential

**Fig. 3:**
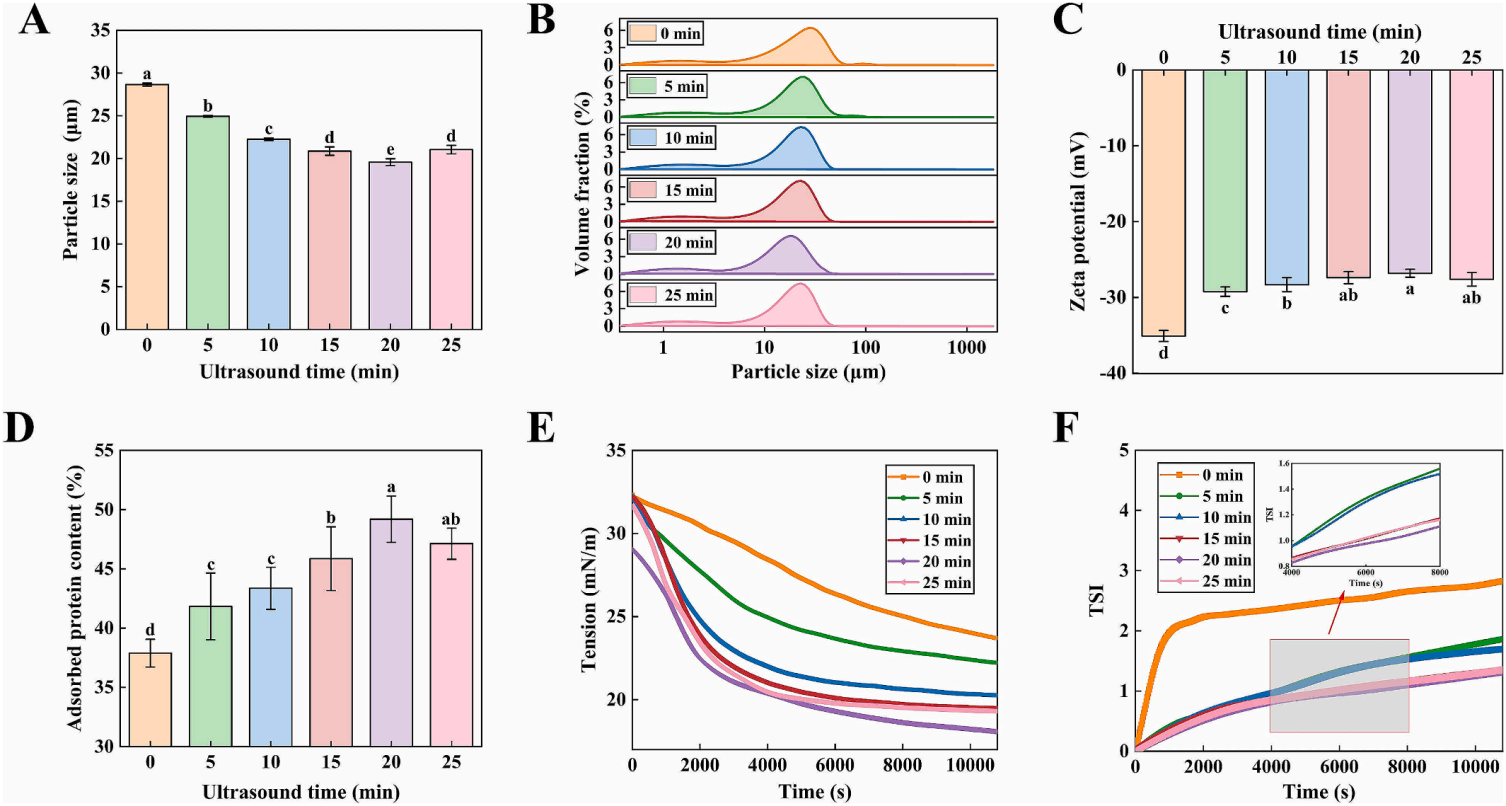
Particle size (A), particle size distribution (B), zeta potential (C), interfacial adsorbed protein content (D), interfacial tension (E) and turbiscan stability index (TSI) (F) of Pickering emulsions stabilized by water-dispersible PSE-like myofibrillar protein microgel particles (MMPs) prepared with different ultrasound times (0, 5, 10, 15, 20, and 25 min). Different letters (a–e) above error bars indicate significant difference (*p* < 0.05) among different samples.

The Pickering emulsion prepared with untreated MMPs was characterized by a comparatively large droplet size (28.7 ± 0.2 μm) and a broad size distribution (Fig. 3A and B). After ultrasonication, the emulsion droplets became smaller, and their size distribution shifted toward smaller sizes with a narrower distribution range, indicating that ultrasound improved emulsion uniformity. Among all treatments, the emulsion prepared with MMPs treated for 20 min showed the smallest droplet size (19.6 ± 0.4 μm). This phenomenon may be related to ultrasound-induced particle refinement, because the size of stabilizing particles directly affects their migration to the oil–water interface, interfacial coverage efficiency, and final emulsion droplet size. Smaller MMPs possess a greater specific surface area and can diffuse more rapidly to oil–water interfaces during emulsification, providing more potential adsorption sites and facilitating interfacial coverage. Consequently, emulsions with smaller droplets and better dispersibility can be obtained [29]. Hussain Badar, et al. [43] also reported similar findings. Notably, a smaller MMP size does not necessarily guarantee better Pickering emulsion stability. Upon extending the ultrasound treatment to 25 min, the emulsion droplet size marginally increased, suggesting that further particle refinement did not further improve emulsification performance. This may be because excessive ultrasound reduced surface hydrophobicity and contact angle, thereby weakening the interfacial adsorption of MMPs at the oil–water interface. Therefore, the slight increase in droplet size in the 25 min group was likely caused by the combined effects of excessive particle refinement and reduced effective interfacial coverage, rather than by particle size alone [23].

The absolute zeta potential of the emulsions initially decreased and then slightly increased with increasing ultrasound treatment time (Fig. 3C). Among all samples, the emulsion stabilized by MMPs treated with ultrasound for 20 min exhibited the lowest absolute zeta potential value (−26.82 ± 0.52 mV), which corresponded to the zeta potential of the corresponding MMPs. This suggests that the stability of this system was not primarily governed by electrostatic repulsion, but was more likely associated with interfacial adsorption, steric hindrance, and the physical barrier provided by the adsorbed MMP layer [44]. In general, a higher absolute surface charge is beneficial for particle dispersion stability. However, in Pickering emulsions, close particle packing at the oil–water interface may be hindered by excessive electrostatic repulsion. Conversely, a moderate lowering of surface charge may facilitate particle association at or near the interface and contribute to the formation of an interfacial particle layer or weak continuous-phase particle network [45]. However, further prolonging the ultrasound time slightly increased the absolute zeta potential while simultaneously increasing the droplet size. This suggests that the slightly enhanced electrostatic repulsion could not compensate for the reduced adsorption capacity caused by decreased surface hydrophobicity and wettability, resulting in weaker interfacial retention and reduced emulsion stabilization.

### 3.7 Pickering emulsion AP content, interfacial tension and TSI

Compared with the non-ultrasonicated group, AP increased significantly from 37.89 ± 1.17% to 49.18 ± 1.95% as the ultrasound time was extended to 20 min, and then receded to 47.12 ± 1.31% at 25 min (Fig. 3D). This indicates that moderate ultrasound treatment promoted MMPs adsorption at the interface, whereas excessive ultrasound did not further improve interfacial coverage. Combined with the above results, the increase in AP was not caused by a single factor, but by the synergistic regulation of particle size and surface properties induced by ultrasound treatment. On the one hand, ultrasound reduced MMP particle size and improved dispersion uniformity, enabling microgel particles to migrate and adsorb more efficiently during emulsification [8]. On the other hand, moderate ultrasound-induced conformational rearrangement increased the exposure of hydrophobic groups and adjusted particle wettability to a range more favorable for interfacial retention, thereby enhancing the interfacial adsorption capacity of MMPs [46]. Together, these changes allowed more MMPs to participate in interfacial coverage and form a more effective particle-stabilized interfacial layer, thereby conferring better tolerance to droplet coalescence and flocculation. In contrast, excessive ultrasound may cause the partial re-embedding or reduced accessibility of hydrophobic sites, thereby weakening the interfacial adsorption capacity of MMPs [47].

Compared with AP content, interfacial tension further reflects the effectiveness of adsorbed particles in reducing interfacial free energy and contributing to interfacial stabilization. Fig. 3E shows that the interfacial tension values of each sample dropped rapidly at first and then leveled off, suggesting that MMPs could translocate to the oil–water interface and subsequently undergo persistent interfacial adsorption and conformational restructuring [48]. The 20 min treatment group exhibited the lowest interfacial tension, approximately 18.08 mN/m. Combined with the maximum AP content, this result suggests that MMPs under this condition exhibited stronger interfacial activity and more favorable adsorption behavior at the interface, thereby producing a lower interfacial free-energy state. In contrast, prolonged ultrasound treatment led to reduced AP content and decreased surface hydrophobicity and wettability, which may weaken the interfacial adsorption capacity and increased instability of the interfacial adsorption layer [44].

The TSI of the non-ultrasonicated group increased rapidly during the initial stage and remained the highest throughout the monitoring period (Fig. 3F), indicating that this emulsion was the most prone to destabilization. This result was consistent with the larger particle and droplet sizes, lower AP content, and higher interfacial tension of the untreated system, suggesting its poor ability to generate a protective layer at the interface. After ultrasound treatment, the marked decrease in TSI values implies that sonication effectively promoted the storage durability of MMP-stabilized Pickering emulsions. Combined with the above results, moderate ultrasound enhanced particle adsorption at the interface by increasing surface hydrophobicity and contact angle, thereby suppressing droplet migration and aggregation. The 15 min and 25 min treatment groups exhibited relatively low and relatively similar trends, with the 20 min group showing the lowest value among them. This suggests that MMPs within this treatment range possessed sufficient interfacial stabilization ability to maintain emulsion stability during short-term monitoring.

### 3.8 Pickering emulsion dynamic rheological characteristics

**Fig. 4:**
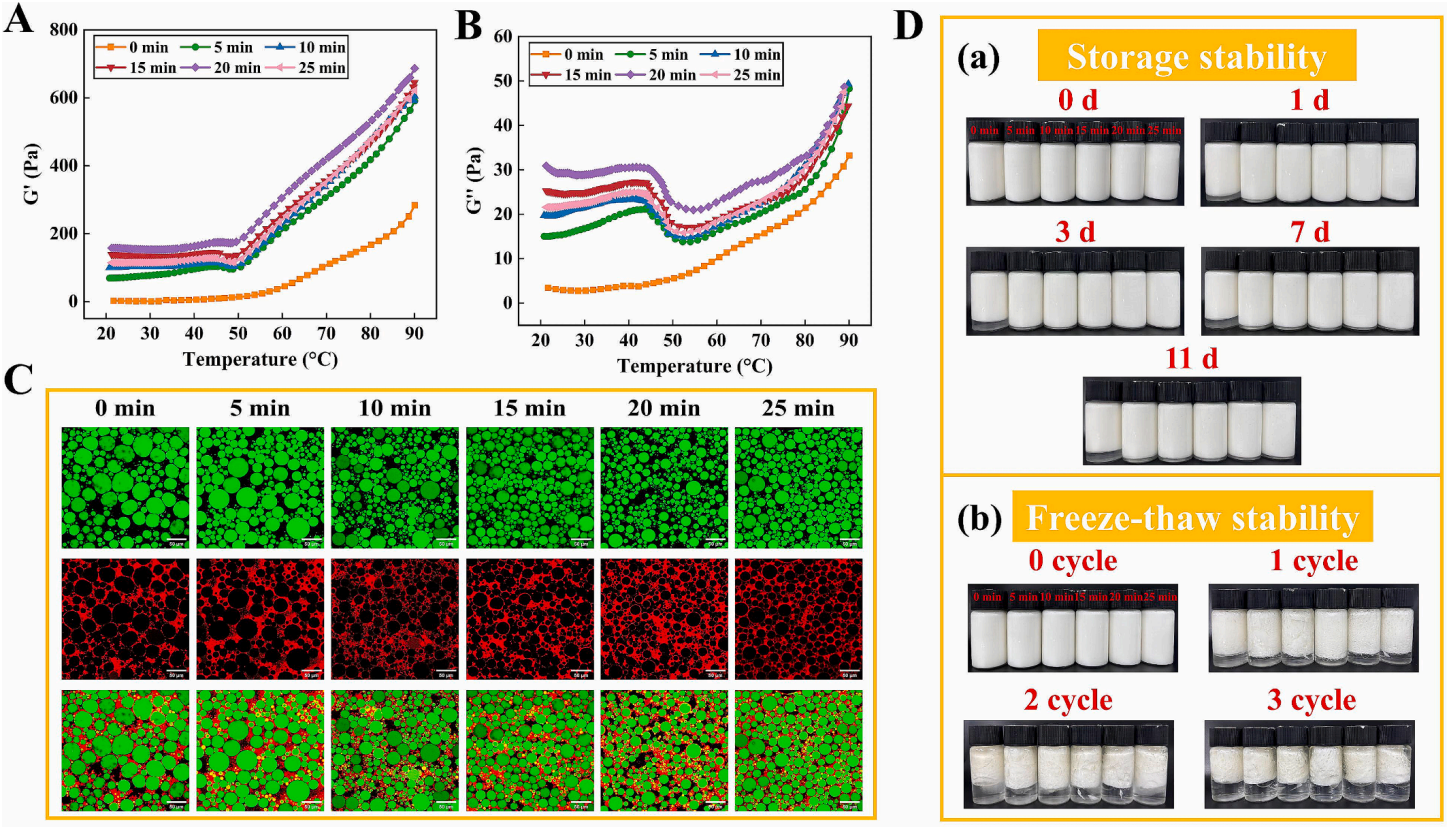
Temperature scan storage modulus (G′) (A), loss modulus (G″) (B), confocal laser scanning microscopy images (CLSM) (C) and storage stability (a), freeze-thaw stability (b) (D) of Pickering emulsions stabilized by water-dispersible PSE-like myofibrillar protein microgel particles (MMPs) prepared with different ultrasound times (0, 5, 10, 15, 20, and 25 min).

The temperature sweep results indicated that the Pickering emulsion system exhibited pronounced heat-induced structural reinforcement. As shown in Fig. 4A and B, both G′ and G″ of the 0 min group remained at relatively low levels, indicating its limited network-forming ability. The ultrasonicated groups exhibited higher G′ and G″ values, with G′ persistently exceeding G″ across the entire tested temperature sweep, suggesting elastic-dominated weak-gel or gel-like behavior. This result indicates that ultrasonicated MMPs improved the structure-building capacity coupled with enhanced thermostability of the Pickering-stabilized systems. At the low-temperature stage, G′ changed only slightly, indicating relatively stable initial structures. Around 45–55 °C, the slight fluctuation in G′ and G″ may be associated with interfacial particle rearrangement and adjustment of weak interactions within the continuous phase [49]. At higher temperatures, G′ increased rapidly, indicating the construction of a stronger emulsion gel scaffold, most likely through the cooperative action of the particle-covered interface, droplet interactions, and the heat-induced continuous protein network [50]. Among all treatments, the G′ and G″ curves of the 15 min and 25 min treatment groups exhibited relatively similar trends throughout the heating process, indicating that the structural evolution behavior of MMPs during heat-induced gelation was similar under these treatment conditions. This may be attributed to the failure of these groups to achieve an optimal balance between particle size and interfacial activity, resulting in emulsion gel networks with comparable structural strength that did not exhibit significant differences in moduli. In contrast, the 20 min group exhibited the highest G′ and G″ throughout heating and showed the greatest final structural strength. This may be attributed to the suitable balance between interfacial adsorption and continuous-phase network formation: MMPs with appropriate size and wettability stabilized smaller droplets, while non-adsorbed MMPs contributed to the heat-induced continuous network.

### 3.9 Pickering emulsion CLSM

As shown in Fig. 4C, oil droplets stained green were dispersed in the aqueous phase and surrounded by a continuous red MMP layer, indicating that interfacial adsorption of MP gel particles led to the creation of typical Pickering O/W emulsions. The untreated MMP-stabilized emulsion exhibited relatively large and unevenly distributed droplets, with some droplets exhibiting irregular shapes. Emulsion droplets exhibited a progressive reduction in size and improved uniformity of dispersion with prolonged ultrasound treatment. In particular, in the 20 min treatment group, the oil droplet surfaces were covered by a continuous red MMP layer, indicating that moderate ultrasound markedly facilitated the interfacial assembly and arrangement of MMPs onto the droplet surface, forming an interfacial film. In contrast, although the 25 min treatment group still maintained good dispersion, compared with the 20 min group, its droplet size was marginally larger, consistent with the droplet size analysis. These microstructural differences may arise from the synergistic regulation of MMP particle structure and interfacial behavior by ultrasound treatment. These microstructural observations further supported the interfacial behavior discussed above. Meanwhile, the rough and irregular morphology of MMPs may increase their contact area with the oil–water interface, thereby facilitating interfacial attachment and coverage [45]. In addition, non-adsorbed gel particles distributed in the continuous phase may physically restrict droplet migration and reduce droplet collision and aggregation [18]. Therefore, the interfacial particle layer, together with the physical restriction provided by continuous-phase particles, helped improve the Pickering emulsion stability.

### 3.10 Pickering emulsion storage stability and freeze-thaw stability

Fig. 4D (a) displays the macroscopic appearance of the emulsions during storage. Phase separation was evident in the untreated emulsion by day 1, and its severity increased with storage duration. This was mainly associated with its larger droplet size and insufficient interfacial coverage. Larger droplets generally have a higher creaming velocity and a greater probability of droplet collision and coalescence, ultimately leading to creaming and phase separation [47]. In contrast, emulsions stabilized by MMPs treated for 20 and 25 min did not show obvious phase separation until day 7. By day 11, the 15–25 min treatment groups showed no obvious visual differences and exhibited relatively high macroscopic stability. This suggests that smaller droplets, a well-covered interface, and the continuous-phase particulate network synergistically impeded drop movement along with coalescence. However, these visual observations only reflect macroscopic stability and are insufficient to distinguish subtle variations in microstructure and interfacial properties across groups.

As shown by the visual appearance after freeze-thaw cycles (Fig. 4D (b)), all emulsions exhibited varying degrees of oil and water separation following the initial freeze-thaw cycle. The untreated group showed the most pronounced separation, whereas the 15 min ultrasound-treated group exhibited relatively less phase separation. Upon completion of three freeze-thaw cycles, severe phase separation was observed in all treatment groups. Freeze-thaw destabilization of emulsions is generally associated with ice crystallization-induced cryoconcentration during freezing. Enlargement of ice crystals compresses the space occupied by droplets and particles, increases droplet contact and aggregation, and weakens the stability of the interfacial film and continuous-phase network, which ultimately appears as oiling-off, water release, and phase separation after thawing [27]. Although ultrasound-induced MMPs improved interfacial film compactness and reinforced continuous-phase structural support, they could not fully prevent the irreversible structural damage caused by repeated freeze-thaw cycles. This likely results from the low ionic strength of the system was insufficient to effectively regulate ice crystal formation and recrystallization, thereby failing to reduce the mechanical damage caused by ice crystals to oil droplets and interfacial films. In addition, MMPs may undergo freeze-induced aggregation during freeze-thaw cycling, reducing their interfacial stabilization ability and limiting their capacity to protect emulsions against ice crystal-induced damage [51]. These findings further suggest that ultrasound enhanced the physical stability of Pickering emulsions, but its enhancement of freeze-thaw stability was limited.

### 3.11 Pickering emulsion gel strength and emulsion stability

**Fig. 5:**
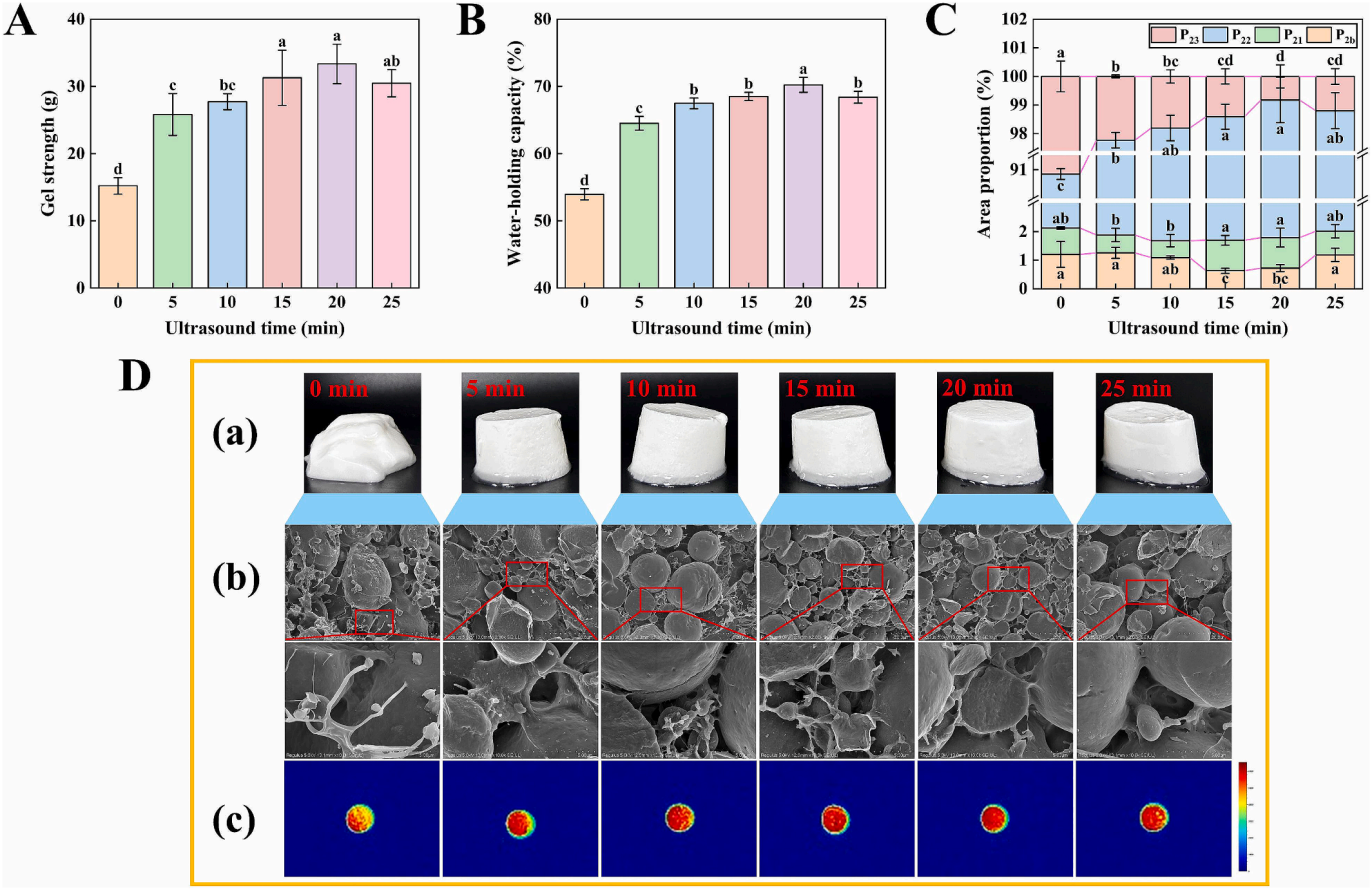
Gel strength (A), water-holding capacity (WHC) (B), low-field nuclear magnetic resonance (LF-NMR) (C) and photographs (a), cryo-scanning electron microscopy (cryo-SEM) (b) and magnetic resonance imaging (MRI) (c) (D) of Pickering emulsion gels stabilized by water-dispersible PSE-like myofibrillar protein microgel particles (MMPs) prepared with different ultrasound times (0, 5, 10, 15, 20, and 25 min). Different letters (a–d) above error bars indicate significant difference (*p* < 0.05) among different samples.

As shown in Fig. 5A, gel strength increased after ultrasound treatment compared with the untreated group, although the 15–25 min treatment groups showed comparable values (*p* > 0.05). A significant improvement in WHC was observed for the treated groups, peaking at 20 min of treatment (Fig. 5B). These results indicate that ultrasound treatment generally enhanced emulsion gel network formation and water retention capacity. This improvement may be associated with the ultrasound-induced optimization of emulsion structure and interfacial–matrix interactions. After moderate ultrasound treatment, the oil droplets became smaller and more uniform in distribution, allowing them to function as active fillers within the heat-induced continuous MMP network. Meanwhile, the MMP adsorption layer on the oil droplet surface may interact with surrounding non-adsorbed MMPs, facilitating the assembly of an droplet–interface–matrix interconnected network coupled with enhanced resistance to external stress [32]. In addition, the denser network structure could narrow water migration channels and restrict free water loss through physical entrapment, thereby improving WHC [52]. In the untreated and excessively ultrasonicated groups (25 min), larger droplets or locally discontinuous structures may have weakened the interfacial–matrix synergy required for network formation, which may explain their relatively lower gel strength and WHC.

### 3.12 Pickering emulsion gel Cryo-SEM, LF-NMR and MRI

Cryo-SEM was used to visualize the network structure, droplet aggregation state in the Pickering emulsion gels. As shown in Fig. 5D (b), all samples formed three-dimensional network structures involving the dispersed oil phase and the continuous protein matrix. These systems mainly exhibited droplet-aggregated gel structures, while the pores of the protein gel network entrapped a small number of droplets, showing features of droplet-filled gels. For droplets separated by relatively long distances, the connection between droplets was mainly mediated by the protein matrix. When adjacent droplets were close to or nearly in contact, continuous protein matrix connections were observed between them, suggesting the formation of bridging-like droplet–matrix connections rather than direct evidence of stable particle bridging [53]. In the non-ultrasonicated control, oil droplets were larger and unevenly distributed, and the gel network was relatively loose with obvious large pores. After ultrasound treatment, the droplet size decreased, the droplet distribution became more uniform, and the distance between droplets was shortened. These changes enabled smaller droplets to function as reinforcing agents within the protein scaffold and facilitated the formation of a more integrated droplet–interface–matrix network. Particularly in the 15–20 min groups, droplets were more uniformly immobilized in the continuous phase, the matrix showed better continuity, and the pore size was relatively smaller, indicating moderate ultrasound improved emulsion gels structural integrity. This finding agrees with observations of Zhang, Liu et al. [27]. Conversely, although the 25 min group still maintained a relatively compact network, some larger droplets or locally discontinuous regions were observed, probably due to weakened synergy of the particle layer with the continuous protein gel matrix after excessive ultrasound treatment [32].

LF-NMR results showed that ultrasound treatment time markedly affected the water distribution in the emulsion gel systems (Fig. 5C). Based on the relaxation time distribution, water in the system could be classified into bound water (P_2b_), weakly bound water (P_21_), immobilized water (P_22_), and free water (P_23_) [30]. Overall, P_22_ was consistently dominant, accounting for more than approximately 85%, indicating that most water was confined within the pores of the protein network, the spaces between oil droplets, and interfacial regions, rather than existing in a fully free state. The relatively low proportion of P_23_ indicated that freely migrating water was limited in the system, suggesting strong overall water-binding capacity of the emulsion gels. With increasing ultrasound treatment time, the proportion of P_2b_ decreased, while P_21_ and P_22_ generally increased first and then decreased, with P_23_ showing the opposite trend. The 15–20 min groups exhibited higher P_21_ and P_22_ proportions and the lowest P_23_ proportion, indicating that moderate ultrasound enhanced water restriction within the gel structure. This observation corroborated the higher WHC and more compact microstructure seen in the ultrasound-treated gels, suggesting that the improved droplet distribution and more continuous protein network helped immobilize water and reduce free water migration. The untreated group showed a relatively lower P_22_ proportion and a higher P_23_ proportion, suggesting a looser gel network and insufficient water restriction, which was corroborated by the poor shape retention and structural collapse observed within the micrographs of the control group (Fig. 5D (a)). After 15–20 min of ultrasound treatment, the samples exhibited a more intact appearance, and the compact network observed in the microstructural images further confirmed that water was more stably retained within the gel network.

MRI results further confirmed this trend. As shown in Fig. 5D (c), MRI visualized the spatial distribution of water based on proton density. The red regions in the images represent stronger resonance signals and higher water content [54]. With increasing ultrasound treatment time, the MRI images showed a marked improvement in water distribution uniformity, particularly in the 20 min sample, indicating better internal structural continuity and a higher degree of restriction on water migration within the gel.

### 3.13 Pickering emulsion gel rheological properties

#### 3.13.1 Steady shear test

**Fig. 6:**
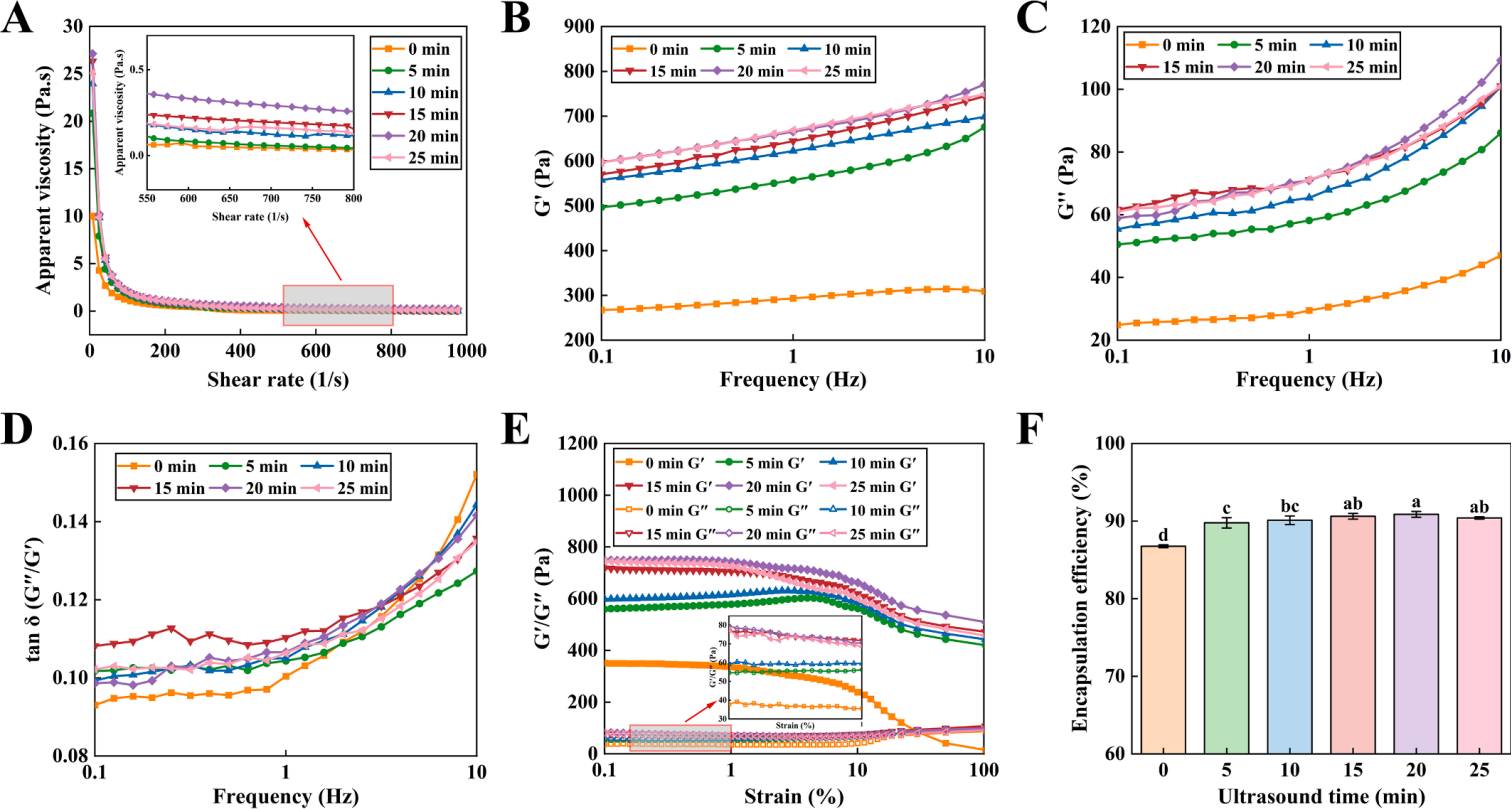
Steady shear test (A), frequency sweep storage modulus (G′) (B), frequency sweep loss modulus (G″) (C), frequency sweep loss tangent (tan δ = G″/ G′) (D), large amplitude oscillation shear (LAOS) (E) and encapsulation efficiency (EE) (F) of Pickering emulsion gels stabilized by water-dispersible PSE-like myofibrillar protein microgel particles (MMPs) prepared with different ultrasound times (0, 5, 10, 15, 20, and 25 min). Different letters (a–d) above error bars indicate significant difference (*p* < 0.05) among different samples.

As shown in Fig. 6A, all samples exhibited typical shear-thinning behavior. In the low-shear-rate region (<100 s^−1^), the apparent viscosity decreased rapidly, suggesting that shear rapidly disrupted the internal network structure constructed by MMPs and the weak interactions among the emulsion gel components. As the shear rate further increased (>200 s^−1^), the decline in viscosity gradually slowed, indicating that the system progressively shifted toward a state dominated by particle orientation and continuous-phase flow [55]. This pseudoplastic behavior is desirable for dysphagia-oriented foods, because relatively high viscosity under static or low-shear conditions helps maintain shape and slow bolus flow, whereas reduced viscosity under oral and swallowing shear facilitates bolus deformation and passage [56]. Ultrasound treatment time markedly affected apparent viscosity. Compared with the non-ultrasonicated group, ultrasound-treated samples showed higher viscosity, which was consistent with the stronger gel network and improved water retention discussed above. In the high-shear-rate region (approximately 500–800 s^−1^), the viscosity differences among the treatment groups decreased, but the 20 min group still maintained a relatively high viscosity, indicating that its structure retained a certain degree of stability under strong shear. For individuals with dysphagia, a moderate increase in shear viscosity may help reduce oropharyngeal flow velocity and extend the time window for airway protection, while avoiding pharyngeal residue caused by excessively high viscosity [57].

#### 3.13.2 Frequency sweep

G′ denotes the solid-like elastic response, while G″ denotes the liquid-like viscous counterpart. The ratio G″/G′, known as tan δ, serves as an indicator: tan δ > 1 indicates viscous behavior, whereas tan δ < 1 indicates elastic behavior [58]. As shown in Fig. 6B and C, all tested samples within the LVR exhibited G′ > G″. Additionally, tan δ values remained below 1 over the full sweep (Fig. 6D). These results indicate the Pickering emulsion gels to be predominantly elastic gel-like in structure, with the systems being elasticity-dominated and exhibiting solid-like behavior. In addition, both G′ and G″ increased progressively over the frequency sweep, indicating that the network exhibited enhanced deformation resistance under higher frequencies, owing to limited structural relaxation. Among all groups, the untreated samples had the smallest G′ and G″ values, reflecting the weakest gel network. After ultrasound treatment, both moduli increased overall and reached relatively high levels in the 20 min and 25 min groups, whose G′ and G″ profiles almost overlapped across the frequency range. These findings suggest that the emulsion gels subjected to the ultrasound treatment exhibited improved network robustness as well as enhanced resistance to strain [50]. Meanwhile, the rheological characteristics of G′ > G″ and tan δ < 1 suggest that these emulsion gels could maintain a certain degree of shape stability during oral and pharyngeal transport, thereby helping to reduce bolus dispersion and pharyngeal residue and consequently lowering the risk of aspiration [59]. However, excessively high moduli may increase the burden of oral processing, which could hinder bolus reformation and smooth swallowing in individuals with dysphagia.

#### 3.13.3 LAOS behavior

Typical elasticity-dominant behavior was observed for the emulsion gel in the low-strain range, as shown by the strain sweep results (Fig. 6E). Within the strain range of approximately 0.1–1%, G′ exceeded G″ for all treated samples, with only minor variations, indicating that the systems remained within the LVR and that the internal gel network could maintain stability under small deformations. As strain increased, G′ gradually decreased whereas G″ increased, suggesting that the externally applied deformation progressively disrupted the spatial architecture of the gel network. With a further increase in strain, the non-sonicated sample showed a crossover between G′ and G″, reflecting a loss of solid-like character and the onset of viscous flow, where the crosslinked structure yielded and underwent macroscopic flow. This phenomenon signifies the disruption of the continuous network together with the attenuation of droplet–droplet associations within the emulsion gel under large deformation [60]. Similar strain-dependent responses have been shown to occur in protein-based composite gel networks [32]. The treatment duration markedly affected the structural strength and deformation resistance of the system. Compared with the 0 min group, all other treatment groups exhibited higher G′ values and maintained linear viscoelastic behavior over a wider strain range, suggesting that appropriate treatment strengthened the structural stability of these emulsion gel systems. Among the samples, the 20 min group showed relatively high G′ and good resistance to large deformation, which may result from the more uniform dispersion of oil droplets and stronger interfacial interactions between droplets and the matrix formed after moderate ultrasound treatment. In contrast, although the 25 min group maintained a relatively high initial modulus, its G′ decreased more rapidly at higher strains, suggesting that excessive ultrasound may weaken network continuity or produce locally discontinuous droplet–matrix structures.

### 3.14 Pickering emulsion gel TPA and IDDSI

The textural properties of gels are important indicators for evaluating their structural characteristics, oral processing behavior, and swallowing suitability. As shown in Table 1, the non-ultrasonicated group could not be effectively measured because of its excessively loose structure, indicating insufficient gel network support. After ultrasound treatment, hardness, springiness, cohesiveness, gumminess, and resilience increased overall, suggesting that the emulsion gels transformed from a loose, poorly structured network into a more stable composite arrangement [61], which validated the gel strength and microstructural results. Hardness increased with prolonged ultrasound treatment and tended to stabilize after 15 min, indicating enhanced resistance to compression. Springiness and resilience remained at relatively high levels after 10 min, suggesting improved shape recovery after deformation. Cohesiveness increased in the 10–20 min groups but slightly decreased at 25 min, indicating that moderate ultrasound strengthened internal structural associations, whereas excessive treatment may weaken local network homogeneity or interfacial–matrix continuity. The trend in gumminess was generally consistent with cohesiveness, further confirming the improved structural integrity and resistance to fragmentation of the ultrasound-treated gels [62]. Overall, these findings suggest that ultrasound treatment enhanced the mechanical robustness as well as the structural integrity of the emulsion gels. From a dysphagia-oriented perspective, suitable texture should balance structural strength and swallowing compliance: insufficient strength may impair bolus integrity, while excessively high hardness or springiness may complicate oral breakdown. Therefore, moderate ultrasound treatment, especially for 15–20 min, achieved a more favorable compromise between structural integrity and swallowability.

**Table 1:** Texture profile analysis (TPA) testing of Pickering emulsion gels stabilized by water-dispersible PSE-like myofibrillar protein microgel particles (MMPs) prepared with different ultrasound times (0, 5, 10, 15, 20, and 25 min).

Sample	Hardness/N	Springiness	Cohesiveness	Gumminess/N	Resilience
0	/	/	/	/	/
5	0.529 ± 0.084^c^	0.614 ± 0.051^b^	0.343 ± 0.009^b^	0.184 ± 0.027^c^	0.107 ± 0.011^b^
10	0.572 ± 0.046^bc^	0.710 ± 0.038^a^	0.368 ± 0.020^a^	0.216 ± 0.018^bc^	0.138 ± 0.012^a^
15	0.639 ± 0.049^ab^	0.731 ± 0.024^a^	0.379 ± 0.013^a^	0.246 ± 0.022^ab^	0.133 ± 0.010^a^
20	0.669 ± 0.052^a^	0.752 ± 0.049^a^	0.381 ± 0.021^a^	0.262 ± 0.024^a^	0.141 ± 0.015^a^
25	0.678 ± 0.029^a^	0.741 ± 0.016^a^	0.359 ± 0.010^ab^	0.246 ± 0.009^ab^	0.134 ± 0.007^a^

Different letters (a–c) within the same column indicate significant difference (*p* < 0.05).

**Fig. 7:**
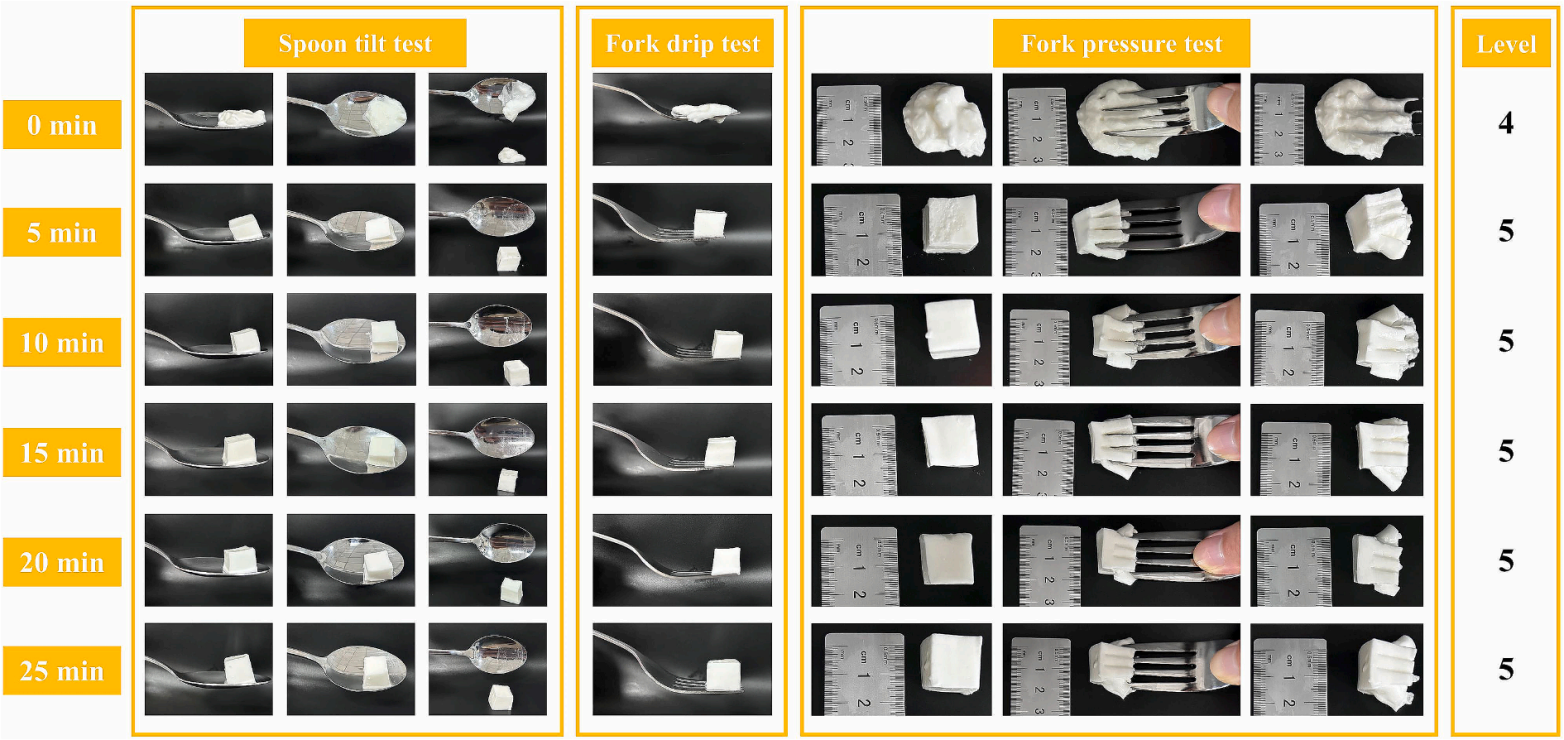
International Dysphagia Diet Standardisation Initiative (IDDSI) of Pickering emulsion gels stabilized by water-dispersible PSE-like myofibrillar protein microgel particles (MMPs) prepared with different ultrasound times (0, 5, 10, 15, 20, and 25 min).

This trend aligned with the IDDSI test findings. These tests revealed differences in swallowing compatibility of the Pickering emulsion gels among samples treated with different ultrasound times (Fig. 7). The untreated group was more prone to shape collapse, edge spreading, or local residue during the spoon tilt and fork drip tests. In contrast, the ultrasonicated groups, especially the 15–25 min groups, maintained their overall shape more effectively in the spoon tilt test and were less likely to undergo uncontrolled dripping in the fork drip test. The spacing between the fork prongs used in the test was 4 mm, which falls within the particle size range considered safe for adults to swallow [62]. The untreated group fractured unevenly after compression, indicating weak bolus retention capacity and poor controllability of its response to applied force. This behavior was closer to the puree-like soft state with limited structural integrity characteristic of IDDSI Level 4. In contrast, the 5–25 min treatment groups exhibited more regular compressive deformation and crushability in the fork pressure test, making them more consistent with the Level 5 (minced and moist) requirements for easily mashed and swallowable foods. In combination with the texture results, these findings further indicate that moderate increases in hardness and gumminess provided the samples with the necessary structural support, whereas the simultaneous enhancement of springiness, cohesiveness, and resilience enabled more controlled deformation and fracture behavior. As a result, the gels could maintain a certain degree of integrity during oral processing without becoming excessively firm and thereby increasing the swallowing burden. In other words, the non-sonicated group tended to exhibit insufficient structural support and weak bolus-forming ability, whereas the 5–25 min groups formed gel states more suitable for oral transport and swallowing initiation. Overall, the improved IDDSI performance suggests that appropriate ultrasonication promoted the transition of Pickering emulsion gels from IDDSI Level 4-like characteristics toward more stable IDDSI Level 5-like characteristics, thereby improving their compatibility with the requirements for dysphagia-oriented foods. The increase in hardness and cohesiveness of the emulsion gel helps explain the improved shape retention and reduced residue observed in the IDDSI tests, while the moderate gel strength and deformation behavior were consistent with the fork pressure results.

### 3.15 Pickering emulsion gel EE

EE reflects the protective capacity of emulsion gels for bioactive compounds during processing, storage, and digestion, as well as their loading ability. Ultrasound treatment markedly improved curcumin EE within the Pickering emulsion gel systems relative to the untreated group (Fig. 6F), increasing EE from 86.75 ± 0.18% to a peak value of 90.87 ± 0.37% at 20 min. Based on the above results, ultrasound treatment may enhance the loading and protective capacity for hydrophobic bioactive compounds by reinforcing the structural integrity of the emulsion gels. Because curcumin is mainly distributed in the oil phase, its retention is closely related to oil droplet stability, interfacial protection, and gel network confinement. Moderate ultrasound treatment improved MMP interfacial adsorption and emulsion gel network formation, which could reduce oil droplet coalescence and oil-phase leakage, thereby helping retain curcumin within the oil droplets and gel matrix [63]. Although curcumin is relatively sensitive to high temperature, the untreated group still exhibited high encapsulation efficiency, indicating that the Pickering emulsion gel system itself possessed good curcumin-loading capacity. Therefore, the improvement induced by ultrasonication should be regarded as a further optimization of retention capacity and interfacial protection efficiency, rather than a fundamental alteration of the loading mechanism. Moreover, EE reached a plateau at ultrasonication times of 15–20 min and decreased slightly at 25 min, suggesting that the system achieved optimal interfacial organization and network construction under 15–20 min ultrasonication. This also indicates that moderate ultrasonication favored the formation of a more effective physical barrier by protein particles, whereas excessive ultrasonication weakened the continuity and compactness of the interfacial film or the particle–matrix synergistic interactions, ultimately limiting further curcumin retention [64]. Overall, the variation in EE primarily arises from the interplay among oil-phase partitioning, interfacial protection, and network confinement in retaining curcumin within the emulsion gel system.

## 4 Conclusion

This study demonstrates that water-dispersible MMPs prepared from ultrasound-regulated PSE-like MP aggregates can improve interfacial functionality and gel properties, providing an effective strategy for developing PSE-like dysphagia-oriented foods. Moderate ultrasonication promoted the disassembly and refinement of MMPs, induced conformational rearrangement, increased surface hydrophobicity, and optimized particle wettability. These changes enhanced MMP adsorption at the oil–water interface, resulting in higher interfacial adsorbed protein content, lower interfacial tension, smaller emulsion droplets, and improved storage stability of Pickering emulsions. After heat-induced gelation, MMP-stabilized oil droplets and the continuous protein matrix jointly formed an integrated droplet–interface–matrix network, thereby improving gel strength, WHC, viscoelasticity, textural stability, and curcumin encapsulation efficiency. Among the treatments, the 20 min group showed the best overall performance by achieving a favorable balance among particle restructuring, interfacial stabilization, gel network formation, and curcumin retention. These findings indicate that ultrasound-regulated MMPs can be used to construct swallowing-friendly Pickering emulsion gels with potential nutrient-delivery capacity. Nevertheless, this study still has certain limitations. The freeze-thaw stability of the system remains limited, and the actual swallowing safety, sensory acceptability, and gastrointestinal digestion and release behavior of the emulsion gels have not yet been systematically investigated. Future studies could improve the freeze-thaw stability of the system through polysaccharide incorporation, construction of composite interfacial films, or ice-crystal regulation strategies, while further integrating evaluations of oral processing, swallowing safety, digestive release, and nutritional bioaccessibility. Overall, this study verifies the feasibility of the colloidal restructuring strategy for developing PSE-like MP-based, swallowing-friendly Pickering emulsion gel foods with potential nutrient-delivery functions.

## CRediT authorship contribution statement

**Linmeng Wang:** Writing – review & editing, Writing – original draft, Validation, Methodology, Investigation, Data curation. **Ke Li:** Writing – review & editing, Writing – original draft, Visualization, Methodology, Funding acquisition, Formal analysis, Conceptualization. **Yu Wang:** Writing – review & editing, Investigation, Formal analysis, Data curation. **Wuchao Ma:** Methodology, Investigation, Formal analysis, Data curation. **Jianan Yan:** Visualization, Methodology, Investigation, Formal analysis. **Ge Han:** Methodology, Investigation, Data curation. **Haitao Wu:** Visualization, Validation, Methodology, Formal analysis. **Yanhong Bai:** Supervision, Resources, Project administration, Funding acquisition, Conceptualization.

## Declaration of competing interest

The authors declare that they have no known competing financial interests or personal relationships that could have appeared to influence the work reported in this paper.
